# Design and Analysis of a New Tuning Fork Structure for Resonant Pressure Sensor

**DOI:** 10.3390/mi7090148

**Published:** 2016-08-24

**Authors:** Xiaodong Sun, Weizheng Yuan, Dayong Qiao, Ming Sun, Sen Ren

**Affiliations:** 1 Key Laboratory of Micro/Nano Systems for Aerospace, Ministry of Education, Northwestern Polytechnical University, Xi’an 710072, China; sunxiaodong@mail.nwpu.edu.cn (X.S.); dyqiao@nwpu.edu.cn (D.Q.); rensen@nwpu.edu.cn (S.R.); 2 LeadMEMS Sci&Tech, Xi’an 710075, China; mercy.sun@leadmems.com

**Keywords:** resonant pressure sensor, tuning fork structure, silicon-on-insulator (SOI), sensitivity, cross-talk

## Abstract

This paper presents a micromachined resonant pressure sensor. The sensor is designed to optimize the sensitivity and reduce the cross-talk between the driving electrodes and sensing electrodes. The relationship between the sensitivity of the sensor and the main design parameters is analyzed both theoretically and numerically. The sensing and driving electrodes are optimized to get both high sensing capacitance and low cross-talk. This sensor is fabricated using a micromachining process based on a silicon-on-insulator (SOI) wafer. An open-loop measurement system and a closed-loop self-oscillation system is employed to measure the characteristics of the sensor. The experiment result shows that the sensor has a pressure sensitivity of about 29 Hz/kPa, a nonlinearity of 0.02%FS, a hysteresis error of 0.05%FS, and a repeatability error of 0.01%FS. The temperature coefficient is less than 2 Hz/°C in the range of −40 to 80 °C and the short-term stability of the sensor is better than 0.005%FS.

## 1. Introduction

The resonant pressure sensor has attracted a lot of attention during the last decades due to its better accuracy and long-time stability [1]. The resonant pressure sensor can be excited and detected in different ways, including piezoelectric excitation and piezoelectric detection, electro thermal excitation and piezoresistive detection, magnetic excitation and magnetic detection, and electrostatic excitation and capacitive detection [2]. Electrostatic excitation combined with capacitive detection is an attractive approach because of the simplicity of the structure and compatibility with micromachining technology [3,4].

A variety of structures designed for resonant pressure sensor with electrostatic excitation and capacitance detection have been put forward [3,4,5,6,7,8,9]. Greenwood developed a resonator comprised with two moving plate [5]. The vibration direction of the resonator is perpendicular with the diaphragm, resulting in a significant energy loss from the resonator to the diaphragm. Welham introduced a laterally driven micro machined resonator for resonant pressure sensor [3]. A lateral mode of oscillation was employed to offer a quality factor insensitive to cavity pressure. The mechanical coupling from the resonator to the diaphragm was reduced as the vibration direction of the resonator is perpendicular with the diaphragm, but there will still be a reaction force transferred to the diaphragm from the resonators as the structure is not designed as dynamic balanced. This will result in a quality factor dependent on the outside conditions.

Ren introduced a micromechanical double-ended tuning fork resonator working in a balanced mode for resonant pressure sensor [6]. The quality factor of this resonator is above 1000 in air and immune to the outside conditions. However, the parasitic capacitance of the sensing and driving electrodes is large and the vibration signal is relatively small, resulting in a large coupling signal from the driving electrode detected in the sensing electrode; and also as the resonator is packaged under atmospheric pressure, the reference pressure of the hermetically packaged air is very susceptible to temperature changes, which will lead a large temperature coefficient of the sensor. 

In this paper, we propose a tuning fork resonator for resonant pressure sensor with low parasitic signal and large vibration signal. The sensitivity of the sensor is optimized by analyzing the deformation of diaphragm and the frequency of the resonator theoretically. The sensor is fabricated by a simple silicon-on-insulator (SOI) microelectromechanical systems (MEMS) process and packaged in a vacuum environment.

## 2. Structure Design 

### 2.1. Structure and Working Principle

**Figure 1:**
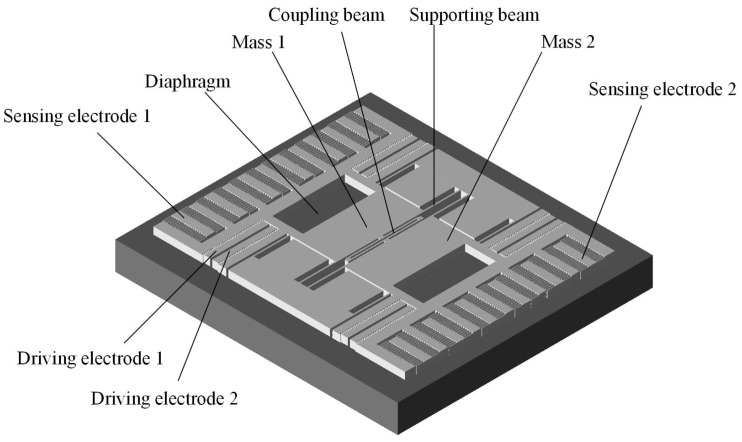
The schematic diagram of the designed silicon resonant pressure sensor.

**Figure 2:**
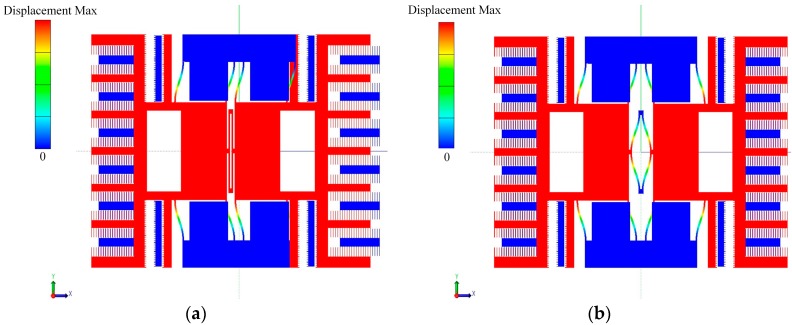
(**a**) The first vibration mode of the resonator; (**b**) The second vibration mode of the resonator.

Figure 1 shows the schematic diagram of the designed silicon resonant pressure sensor which is based on a SOI wafer. This structure is mainly composed of a resonator that is fabricated in the top device layer and a pressure sensitive diaphragm fabricated in the substrate handle layer. Several anchors were formed using the buried oxide layer to connect the resonator and diaphragm. The diaphragm deformation induced by external pressure imposed on the bottom surface of the diaphragm is transferred to the resonator by the anchors, resulting in a change of the natural frequency of the resonator. By detecting the frequency change, the external pressure can be obtained indirectly. The resonator is comprised by two masses moving in opposite direction to reduce the energy loss. Every mass was supported by four beams. The shape of the first and second vibration mode of the resonator by simulation is shown in Figure 2, in which the second mode is the working mode and the first is a parasitic mode. The deflection of the resonator in Figure 2 has no dimensions and only shows the shape of the vibration mode. The parts with red color have a maximum deflection relatively while the parts with blue color have no deflection. A coupling beam between the two masses is used to separate the two modes in frequency domain. Two pairs of driving electrodes and a sensing electrode are placed within each mass frame. The driving and sensing electrodes are separated by the mass frame to reduce the coupling capacitor between them. A differential driving electrodes scheme is implemented for each pair of driving electrodes to suppress the cross-talk between the driving electrodes and sensing electrodes. The sensing electrodes for the two mass frames are configured in this way to achieve a low coupling using a differential sensing technique. The drive electrodes are designed as the area-varying comb capacitors to get a linear driving force and the sense electrodes are designed to be the gap-varying comb capacitors to get a larger sensing signal. There are 216 fingers for each side of sensing capacitor with the length of 80 μm and overlap of 75 μm. The minor gap of the sensing capacitors is 2 μm and the larger gap is 4 μm. Assuming that the vibrating amplitude of the resonator is 0.1 μm, the sensing capacitors can be calculated as 35 fF by simulation.

### 2.2. Analysis of the Sensitivity

The sensitivity of the sensor can be calculated through a detailed analysis of the diaphragm and resonator under a specific pressure.

**Figure 3:**
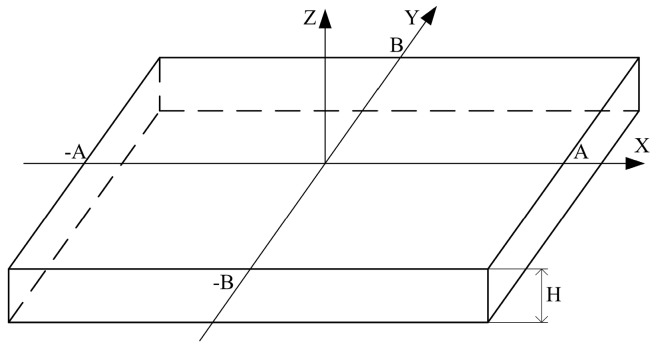
The schematic drawing of the pressure sensitive diaphragm.

Figure 3 shows the schematic drawing of the pressure sensitive diaphragm. When a pressure *P* is imposed on the bottom surface, the differential equation representing the deformation of the diaphragm can be expressed as [10]:

(1) $$\int_{-A}^{A}\int_{-B}^{B}\left[D\left(\frac{\partial^{4}t}{\partial x^{4}}+2\frac{\partial^{4}t}{\partial x^{2}\partial y^{2}}+\frac{\partial^{4}t}{\partial y^{4}}\right)-P\right]\delta tdxdy=0$$

where *A* and *B* is one half of the length along *X* and *Y* axis, t is the deflection of the corresponding point in diaphragm, *D* is the bending rigidity of the diaphragm that can be expressed as:

(2) $$D=\frac{EH^{3}}{12\left(1-\nu^{2}\right)}$$

*E* is the modulus of elasticity, *H* is the thickness of the diaphragm in *Z* axis, and *ν* is the Poisson ratio.

As the membrane is fixed to the frame in surroundings, the boundary condition of Equation (1) is:

$$x=A\text{ and }-A:t=0\;\frac{\partial t}{\partial x}=0$$

$$y=B\text{ and }-B:t=0\;\frac{\partial t}{\partial y}=0$$

The exact solution of Equation (1) cannot be solved but an approximate solution is [10]:

(3) $$t=C{\left(x^{2}-A^{2}\right)}^{2}{\left(y^{2}-B^{2}\right)}^{2}$$

where *C* can be solved by energy method as [10]:

(4) $$C=\frac{49P}{128D\left(7A^{4}+7B^{4}+4A^{2}B^{2}\right)}$$

The displacement in the *Y* axis direction for the upper surface of the diaphragm can be expressed as [10]:

(5) $$\mu =-\frac{\partial t}{\partial y}z$$

where *z* is one half of the thickness of the diaphragm along the *Z* axis and μ is the displacement.

The above Expression (5) can be transformed to the following by using Equation (3) to replace *t*:

(6) $$\mu =-4Cyz{\left(x^{2}-A^{2}\right)}^{2}\left(y^{2}-B^{2}\right)$$

The anchor of the resonator should be distributed along *X* or *Y* axis in the upper surface of the diaphragm in order to suppress the undesired deformation of the resonator. Assuming the anchors are distributed along *Y* axis, the displacement in the *Y* axis direction for the point in *Y* axis can be expressed linearly as:

(7) $$\mu =-4CyzA^{4}\left(y^{2}-B^{2}\right)$$

**Figure 4:**
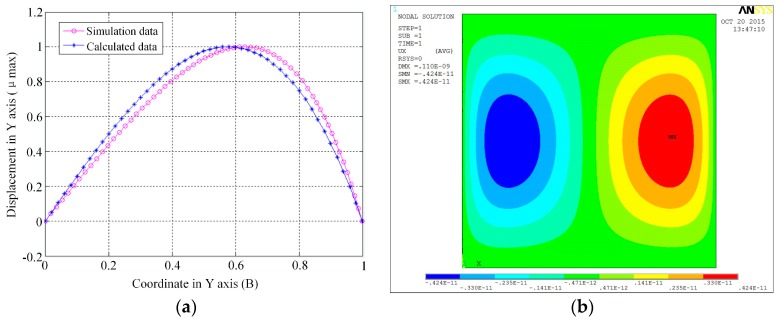
(**a**) The displacement calculated by Equation (7) and simulation; (**b**) The graph of the displacement in *Y* axis direction by simulation.

Figure 4 shows the displacement in *Y* axis direction for the point in the *Y* axis calculated from Equation (7) and finite element simulation. It shows that the results calculated from Equation (7) agree well with the results by finite element simulation and the point in the *Y* axis with the maximum displacement in *Y* axis direction is located in *y* = ±0.6 B. So, in order to increase the sensitivity of the sensor, the anchors for the resonator should be placed in *y* = ±0.6 B.

The frequency of the resonator can be expressed as $\omega =\sqrt{\frac{K_{\text{eff}}}{M}}$, where *K*_eff_ is the effective stiffness of the beams and *M* is the mass of the resonator. According to the structure of the resonator, the effective stiffness *K*_eff_ can be calculated as:

(8) $$K_{\text{eff}}=K_{1}+K_{2}+K_{3}$$

where *K*_1_ is the stiffness of the supporting beams for the resonator connecting to the anchors, *K*_2_ is the stiffness of the coupling beams connecting the two masses, *K*_3_ is the increased stiffness of the supporting beams induced by the deformation of the diaphragm. 

**Figure 5:**
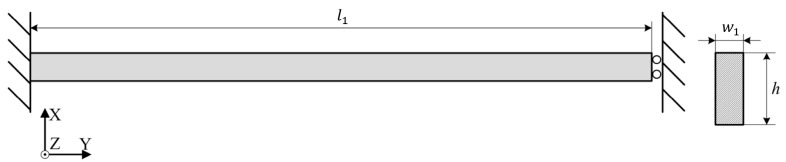
The schematic diaphragm of the supporting beam.

The schematic diaphragm of the four supporting beams for each mass in the resonator can be shown in Figure 5. One end of the beam only has the freedom of *X* axis and the other end is fixed. So the stiffness of the beams for the direction of *X* axis can be expressed as [10]:

(9) $$K_{1}=\frac{4Ehw_{1}^{3}}{l_{1}^{3}}$$

where $l_{1}$, $w_{1}$, and h is the length, width, and thickness of the supporting beams, respectively.

The two masses of the resonator move in opposite direction, so the coupling beams have a similar deformation to the supporting beams. The stiffness of the coupling beams can be expressed as [10]:

(10) $$K_{2}=\frac{2Ehw_{2}^{3}}{l_{2}^{3}}$$

where $l_{1}$, $w_{1}$, and h is the length, width and thickness of the supporting beams, respectively.

*K*_3_ can be derived by energy method as [11,12]:

(11) $$K_{3}=\frac{24Ehw_{1}\Delta l_{1}}{5l_{1}^{2}}$$

where $\Delta l_{1}\;$ is the deformation of the supporting beam in axial direction due to the deformation of the diaphragm. Substituting expressions of *K*_1_, *K*_2_, and *K*_3_ into Equation (8), the natural frequency of the resonator can be expressed as:

(12) $$f=\frac{1}{2\pi }\sqrt{\frac{K_{\text{eff}}}{M}}=\frac{1}{2\pi }\sqrt{\frac{\frac{4Ehw_{1}^{3}}{l_{1}^{3}}+\frac{2Ehw_{2}^{3}}{l_{2}^{3}}+\frac{24Ehw_{1}\Delta l_{1}}{5l_{1}^{2}}}{M}}$$

so the sensitivity of the natural frequency respect to the axial deformation is:

(13) $$S=\frac{df}{d\Delta l_{1}}=\frac{6Ehw_{1}}{5\pi l_{1}^{2}}\sqrt{\frac{1}{M\left(\frac{4Ehw_{1}^{3}}{l_{1}^{3}}+\frac{2Ehw_{2}^{3}}{l_{2}^{3}}+\frac{24Ehw_{1}\Delta l_{1}}{5l_{1}^{2}}\right)}}$$

It can be seen from Equation (13) that the sensitivity of the natural frequency with respect to the axial deformation is mainly related to the dimensions of the supporting beams and coupling beams. In order to increase the sensitivity, the stiffness of the coupling beams should not be large and the dimensions of the supporting beams should be small. On the other hand, a small stiffness of the coupling beams will lead to a small frequency deviation of the parasitic mode and the working mode and a small dimensions means a large relative deviation for fabrication.

The parameters of the sensor used in this work is shown in Table 1. The frequency of the first and second mode is 36 kHz and 42 kHz under these parameters, so the crosstalk between different modes can be suppressed. The sensitivity of the sensor is about 35 Hz/kPa by Equation (13). The minimum size of beams is 6 μm and a better consistency can be guaranteed by our fabricating process.

## 3. Experiment

**Figure 6:**
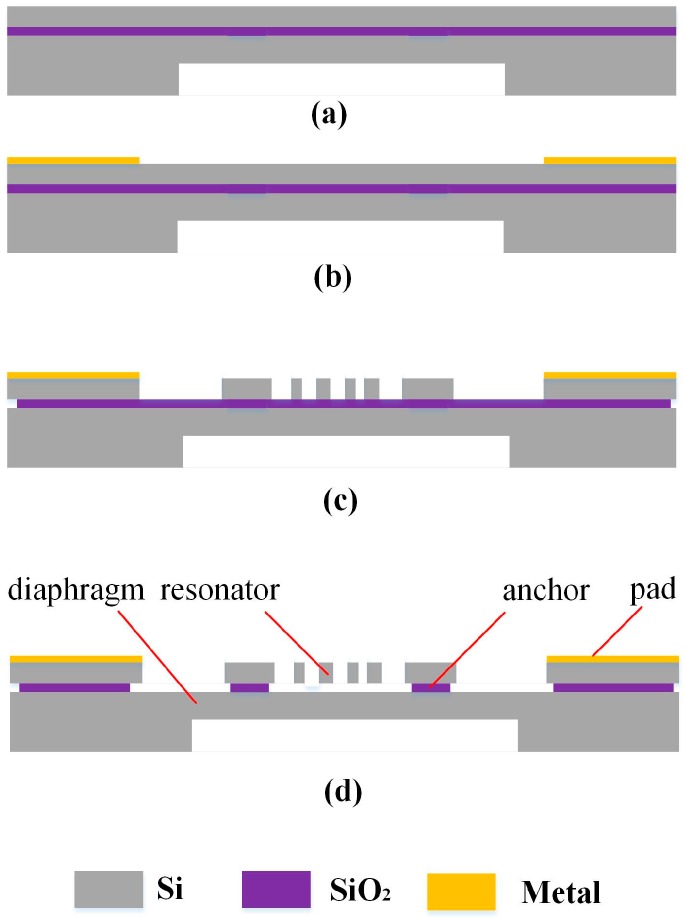
The fabricating process of the sensor. (**a**) The handle layer of a SOI wafer is etched by DRIE; (**b**) The metal Au is deposited selectively in the surface of device layer to form pads; (**c**) The device layer is etched to the buried oxide layer by DRIE to form the resonator structure; (**d**) The exposed buried oxide layer is etched by hydrogen fluoride.

This sensor is fabricated using a micromachining process based on a SOI wafer, as shown in Figure 6. Firstly, the handle layer of a SOI wafer is selectively etched by deep reactive ion etching (DRIE); the remaining thickness of the handle layer in the etched area is precisely controlled so as to form the diaphragm. Then the metal Au is deposited selectively in the surface of device layer to form pads. Thirdly, the device layer is etched to the buried oxide layer by DRIE to form the resonator structure. Fourthly, the exposed buried oxide layer is etched by hydrogen fluoride (HF) and then the resonator structure is released.

**Figure 7:**
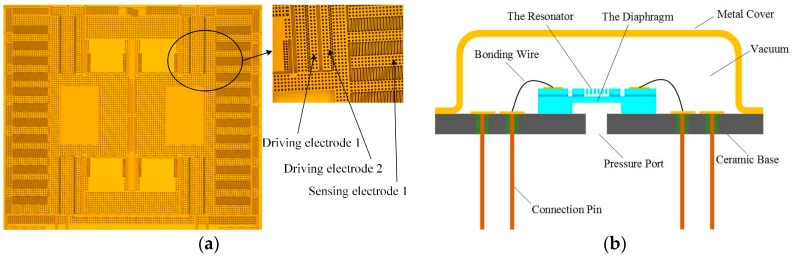
(**a**) The picture of the fabricated resonator; (**b**) The schematic drawing of the sensor package.

The picture of the fabricated resonator is shown in Figure 7a, where the driving and sensing electrodes are enlarged. Figure 7b shows the schematic drawing of the sensor package. The resonator is encapsulated in a ceramic package under a vacuum environment to get a high quality factor. The upper surface of the pressure sensitive diaphragm is contacted to the vacuum environment while the lower surface is contacted to the measured pressure. The pressure difference of the two surfaces will lead to a deformation of the diaphragm, which will results in a frequency variation of the resonator.

**Figure 8:**
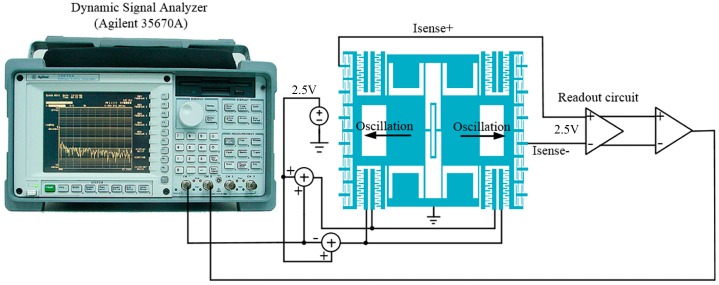
The open-loop measurement system for the sensor.

**Figure 9:**
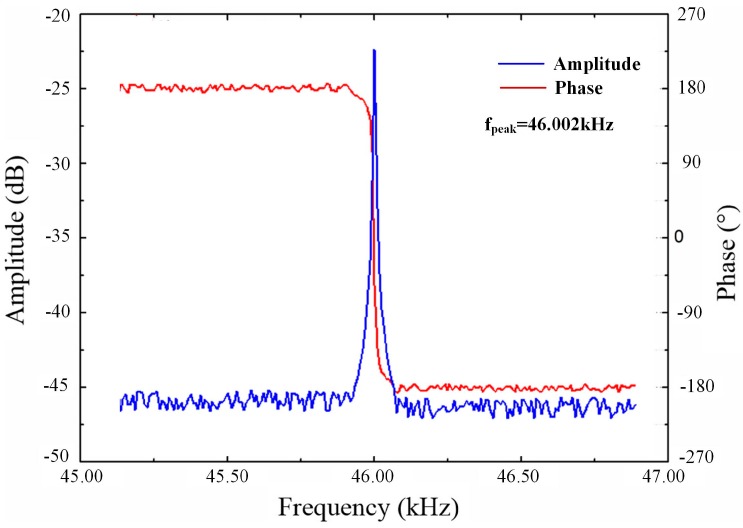
The resonance characteristics of the sensor.

In order to measure the performances of the resonator, an open-loop system is implemented as shown in Figure 8. A dynamic signal analyzer Agilent 35670A (Agilent, Santa Clara, CA, USA) is used to produce an AC voltage. This AC voltage should combined with a DC voltage to produce a driving force for the resonator. In this paper, the DC bias voltage on driving electrodes in Figure 8 is set as 2.5 V, which is the common-mode voltage of the circuit by a 5 V supply. In order to measure the changing of the sensing capacitance, a DC voltage should also be applied on the sensing electrodes, which is also set as 2.5 V. The output voltage signal of readout circuit is fed back to the dynamic signal analyzer and compared with the driving signal. The resonator is encapsulated in a vacuum environment below 10 Pa and the lower surface of the pressure sensitive diaphragm is contacted to the atmosphere (97.5 kPa). The resonance characteristics of the resonator measured by this system is shown in Figure 9. It can be shown that the phase-frequency curve is smooth and has no anti-resonance phenomenon which can be caused by cross-talk [13]. The resonant frequency of the resonator is 46.002 kHz, where the output signal has a maximum amplitude, and the quality factor is about 10,000. The resonator can hardly vibrate when the frequency of the driving voltage has a large deviation with respect to the resonant frequency of the resonator, so the output signal for that frequency is mainly comes from the driving crosstalk signal, which has a much smaller amplitude than the peak value in Figure 9. So the crosstalk signal is much smaller than the vibration signal in the resonant frequency and has little effect to the vibration of the resonator.

**Figure 10:**
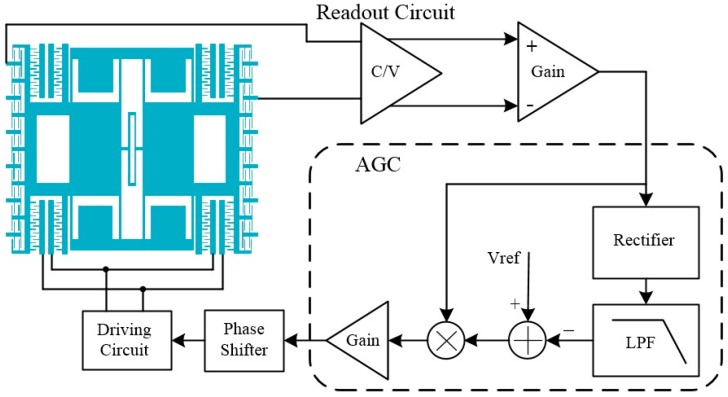
The closed-loop self-oscillation system for the sensor. V_ref_ is the reference voltage for amplitude and LPF is the low-pass filter.

**Figure 11:**
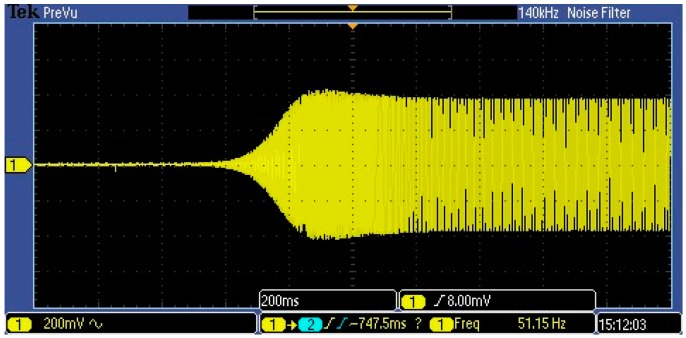
The start-up transient signal of the closed-loop system for the sensor.

A closed-loop self-oscillation system is put forward for the resonator, as shown in Figure 10. A readout circuit is used to detect the vibration signal of the resonator and an automatic gain control (AGC) circuit is employed to control the resonant amplitude of the resonator [14]. A phase shifter is used to compensate the small phase shift of the circuit due to some non-ideal factors. The start-up transient signal of the closed-loop system is shown in Figure 11. It can be seen that the self-oscillation system for the sensor works well and the setting time is less than 300 ms.

**Figure 12:**
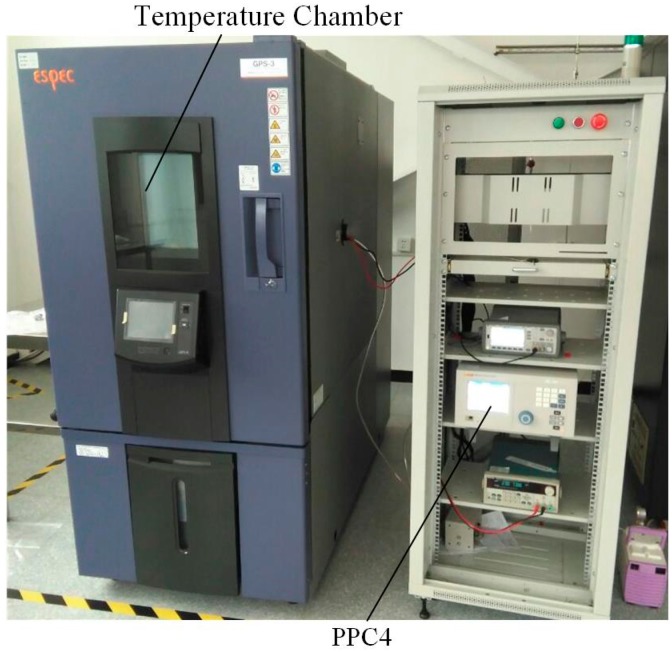
The calibration system for the sensor.

**Figure 13:**
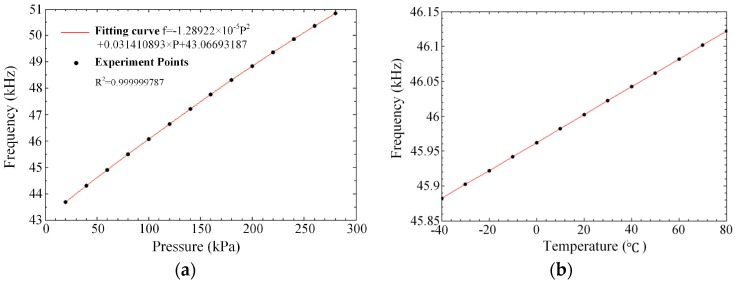
(**a**) The resonant frequency of the sensor under different pressure; (**b**) The resonant frequency of the sensor under different temperature.

A calibration system is then put forward to measure the static performance of the sensor, as shown in Figure 12. A Fluke PPC4 precision pressure controller (Fluke, Everett, WA, USA) is used to supply a precise pressure for the sensor and a temperature chamber is used to keep a constant temperature environment for the sensor. The resonant frequency of the sensor under different pressure in 20 °C is measured by this calibration system and the fitting curve by the results is shown in Figure 13a. The measured data shows that the sensor has a pressure sensitivity of about 29 Hz/kPa, a nonlinearity of 0.02%FS, a hysteresis error of 0.05%FS, and a repeatability error of 0.01%FS. The sensitivity of the sensor has a deviation with the value calculated by theory due to the calculation error and fabricating process deviation. The hysteresis error is not satisfactory here because the package of the sensor deforms and the stress transmitted to the sensor. The resonant frequency under different temperatures is shown in Figure 13b and the temperature sensitivity of the resonator is less than 2 Hz/°C.

**Figure 14:**
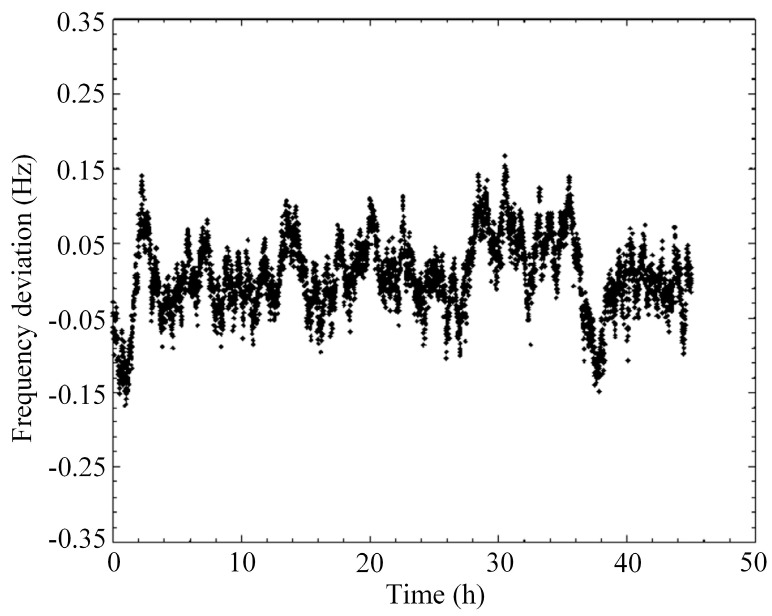
The resonant frequency of the sensor in different time.

A short-term stability of the sensor is tested under a constant pressure of 100 kPa and a constant temperature of 20 °C. The result is shown in Figure 14 and it can be seen that the frequency deviation of the sensor in two days is less than 0.3 Hz, so the short-term stability of the sensor is better than 0.005%FS.

## 4. Discussion

A new designed structure with optimized sensitivity and low parasitic feedthrough is put forward in this paper; the sensitivity of the sensor is analyzed theoretically. After elaborating the design, the sensor is fabricated using a micromachining process based on a SOI wafer. The quality factor of the resonator is about 10000 when encapsulated in a vacuum environment below 10 Pa. The experimental results shows that the crosstalk signal from driving electrodes is very small with respect to the vibration signal of the resonator and the sensor has a pressure sensitivity of about 29 Hz/kPa, a nonlinearity of 0.02%FS, a hysteresis error of 0.05%FS, and a repeatability error of 0.01%FS. The temperature coefficient is less than 2 Hz/°C in the range of −40 to 80 °C and the short-term stability of the sensor is better than 0.01%FS. Further studies are currently underway to improve the accuracy of the resonant pressure sensor and also the long-term stability of the sensor will be characterized which is closely related to the package stress.

## Figures and Tables

**Table 1:** The main designed parameters of the sensor

Parameter	Value
Diaphragm size	1.8 mm × 1.8 mm × 40 μm
Length of supporting beam	150 μm
Width of supporting beam	6 μm
Length of coupling beam	250 μm
Width of coupling beam	9 μm
Device layer thickness	50 μm
Stiffness of the supporting beam	2.16 × 10^3^ N/m
Stiffness of the coupling beam	0.79 × 10^3^ N/m
Mass of the resonator	4.24 × 10^−5^ g
